# Post-stroke outcome prediction based on lesion-derived features

**DOI:** 10.1016/j.nicl.2025.103747

**Published:** 2025-01-31

**Authors:** Maedeh Khalilian, Olivier Godefroy, Martine Roussel, Amir Mousavi, Ardalan Aarabi

**Affiliations:** a Laboratory of Functional Neuroscience and Pathologies (UR UPJV 4559), University Research Center (CURS), University of Picardy Jules Verne, Amiens, France; b Faculty of Medicine, University of Picardy Jules Verne, Amiens, France; c Neurology Department, Amiens University Hospital, Amiens, France

**Keywords:** Lesion, Structural disconnection map, Network connectivity, Stroke

## Abstract

Stroke-induced deficits result from both focal structural damage and widespread network disruption. This study investigated whether simulated measures of network disruption, derived from structural lesions, could predict functional impairments in stroke patients. We extracted four lesion-derived feature sets: lesion masks, probabilistic structural disconnection maps (pSDMs), structural and indirectly estimated functional connectivity strengths between brain regions, and topological properties of functional and structural brain networks to predict motor, executive, and processing speed deficits in 340 S patients, employing PCA-based ridge regression with leave-one-out cross validation.

The findings revealed that both structural disconnection map patterns and lesion masks were strong predictors of functional deficits. Lesion masks exhibited superior predictive performance relative to unthresholded pSDMs. Furthermore, applying a probability threshold to the pSDMs − retaining only disconnections present in a sufficient proportion of healthy subjects − significantly improved predictive performance. For motor deficits, thresholded SDMs (tSDMs) with thresholds of 0.9 and 0.5 produced the highest R^2^ values, 0.95 for left motor deficits and 0.69 for right motor deficits, respectively. In the case of executive function and processing speed, the highest R^2^ values were 0.58 and 0.64, achieved with tSDM thresholds of 0.3 and 0.5, respectively. Connectome-based features exhibited lower R^2^ values, with structural connection strength alterations showing stronger associations with post-stroke scores compared to changes in functional connectivity. Nodal parameters (degree and clustering coefficient) had lower explanatory power than the SDM features and lesion masks.

Our findings underscore the effectiveness of lesion masks and thresholded SDMs in predicting post-stroke deficits. This study contributes to the growing body of evidence supporting the reliability of simulated structural networks as a complementary approach to lesion patterns and structural disconnection in predicting post-stroke outcomes.

## Highlights

• Lesion masks and structural disconnection maps (SDMs) were strong predictors of motor and cognitive deficits.
• Thresholded SDMs outperformed unthresholded SDMs, and lesion masks had higher predictive power than unthresholded SDMs.
• Structural connectivity predicted outcomes as effectively as lesion masks; functional connectivity was less predictive.
• Nodal parameters of brain networks showed lower predictive capability than lesion masks and SDMs.

## 1 Introduction

Post-stroke impairments can arise from both structural damage at the site of injury and broader network dysfunction, often resulting from structural and functional disconnection (Corbetta et al., 2005, Salvalaggio et al., 2020, Siegel et al., 2016, Thiebaut de Schotten et al., 2020). Over the past decade, voxelwise lesion-symptom mapping has emerged as a key method for studying the relationship between focal lesions and functional deficits in stroke patients (Arnoux et al., 2018, Bates et al., 2003). This approach has shown that lesion location and volume can account for 10 % to 35 % of the variance in motor and cognitive performance (Arnoux et al., 2018, Ouin et al., 2022, Puy, L., Barbay, M., Roussel, M., Canaple, S., Lamy, C., Arnoux, A., Leclercq, C., Mas, J.-L., Tasseel-Ponche, S., Constans, J.-M., Godefroy, O., GRECogVASC Study Group et al., 2018, Weaver et al., 2021). More recently, “dysconnectome” approaches have been developed to better understand how focal lesions, particularly in strategic brain regions, disrupt macroscale structural and functional brain connectivity, leading to cognitive and sensory-motor deficits (Foulon et al., 2018, Fox, 2018, Thiebaut de Schotten et al., 2020). These methods use indirect estimates of structural disconnection derived from diffusion imaging data in healthy populations to generate voxel-based disconnection probability maps (Foulon et al., 2018, Fox, 2018), which estimate lesion-caused white matter damage. These maps are then used to predict functional deficits (Salvalaggio et al., 2020, Siegel et al., 2016) and explore the association between functional impairments and structural disconnections in white matter pathways (Boes et al., 2015, Carter et al., 2010, Ivanova et al., 2021, Schaechter et al., 2009, Siegel et al., 2016, Stinear et al., 2007, Umarova et al., 2017).

Studies show that structural disconnections, indirectly estimated from diffusion imaging in healthy subjects, could explain 16 %–58 % of variance in motor, language, spatial attention, and spatial memory performance but less than 6 % in verbal memory (Salvalaggio et al., 2020). These findings underscore the variability in the extent to which structural lesions can predict different functional outcomes. Specifically, motor performance is more consistently associated with focal damage to specific white matter tracts, such as the corticospinal tract (CST) (Byblow et al., 2015, Cho et al., 2007, Godefroy et al., 1998, Kassubek et al., 2005, Lindenberg et al., 2010, Zhu et al., 2010). In contrast, cognitive domains such as executive function and processing speed rely on distributed and less localized neural networks, which makes their prediction inherently more complex (Byblow et al., 2015, Cho et al., 2007, Godefroy et al., 1998, Kassubek et al., 2005, Lindenberg et al., 2010, Zhu et al., 2010). A systematic review by Boyd et al. (2017) identified consistent associations between white matter hyperintensities, global brain atrophy, and post-stroke cognitive impairment, including deficits in executive function (Werring et al., 2004). Similarly, Ferris et al. (2022) examined MRI markers in older adults and chronic stroke patients, demonstrating that regional diffusion tensor imaging metrics (with *R*^2^ = 0.54) outperformed whole-brain lesion volumes (with *R*^2^ = 0.25) in predicting cognitive performance. Despite this, the study concluded that these imaging markers alone are insufficient for accurate predictions, emphasizing the need for multimodal approaches that incorporate additional biomarkers and larger, more diverse samples (Tahmi et al., 2022).

In a recent study, Rivier et al. (2023) used a brain dysconnectome approach, embedding each patient’s lesion within the healthy connectome to construct structural brain graphs. Incorporating brain connectivity metrics into predictive models alongside standard features—such as age, initial motor impairment, lesion volume, and CST asymmetry—significantly improved motor recovery prediction accuracy, raising it from 38 % to 68 %. Similarly, Kuceyeski et al. (2016) showed that models leveraging structural connectome disruption metrics outperformed those based solely on lesion volume in predicting cognitive scores, achieving an *R*^2^ = 0.56 compared to *R*^2^ = 0.37. Their study highlighted that increasing the anatomical specificity of disconnection metrics does not always enhance model accuracy, likely due to the statistical trade-offs introduced by the higher dimensionality of the data.

Resting-state functional connectivity (FC) disruptions have been shown to predict functional deficits in visual and verbal memory more effectively than lesion-derived structural disconnection, explaining 40–60 % of the variability in behavioral scores during the acute stroke stage, even in structurally intact regions (Carter et al., 2010, Salvalaggio et al., 2020, Siegel et al., 2016). FMRI data from stroke patients further demonstrate that FC alterations can distinguish between favorable and unfavorable cognitive recovery (Siegel et al., 2018). Liang et al. (2021) demonstrated that stroke impairment prediction improves significantly when brain network disruptions are considered alongside lesion characteristics. Using machine learning on resting-state FC data, they predicted post-stroke somatosensory function with a correlation coefficient of 0.54. Similarly, Puig et al. (2018) reported that integrating FC measures with clinical scores enhanced the prediction accuracy of 90-day outcomes from 84.2 % to 94.7 %, emphasizing the added value of functional connectivity metrics in prognostic modeling. In contrast, Zhao et al. (2022) found that structural and functional connectivity models did not provide additional predictive value beyond lesion models for aphasia-related functions. This might be due to the redundancy of information across lesion location, structural disconnection features, and connectivity profiles in predicting functional deficits. Another contributing factor could be the curse of dimensionality, where a high number of features contrasts with a small sample size, particularly in predictions based on lesion or disconnection maps (Zhao et al., 2022).

To enhance the prediction of post-stroke functional outcomes, we systematically evaluated the predictive power of lesion masks, structural disconnection maps, and lesion-derived network properties across motor, executive function, and processing speed domains, which differ significantly in their neuroanatomical specificity. Motor performance is closely linked to focal structural damage, while executive function and processing speed are influenced by broader network-level disruptions. Employing a dysconnectome approach, we simulated fiber tracking damage using high-resolution diffusion-weighted imaging (DWI) data from a large, healthy aging population to generate probabilistic disconnection maps for stroke patients.

Lesion-derived structural and functional graphs were incorporated based on emerging evidence that functional deficits result not only from localized brain damage but also from widespread disruptions in network connectivity. Graph-theoretical measures, capturing both global and local changes in network topology, were included as complementary predictors alongside lesion- and disconnection-based metrics, whose relative efficacy has been insufficiently explored in previous research. Additionally, we investigated how simulated and indirect functional connectivity measures contribute to the prediction of functional outcomes. To ensure robust and unbiased comparisons, we employed principal component analysis (PCA)-based ridge regression combined with leave-one-out cross-validation, minimizing overfitting while systematically evaluating the predictive performance of each approach.

## 2 Materials and methods

### 2.1 Patient cohort and clinical Assessment

We used an imaging dataset from the Groupe de Réflexion pour l’Évaluation Cognitive Vasculaire (GRECogVASC) cohort, comprising 340 S patients (207 males, 133 females; mean age: 63.9 ± 10.5 years, range: 40–81) (Godefroy et al., 2012) assessed in Amiens University hospital (Puy et al., 2018). The dataset was collected following the Helsinki Declaration and was approved by the regional investigational review board (Comité de Protection de Personnes Nord-Ouest II, NCT01339195), with written informed consent obtained from each patient. Inclusion criteria were hospitalization within 30 days of acute cerebral infarction or hemorrhage, initial positive imaging, and no prior conditions affecting cognition (Barbay et al., 2018). Exclusion criteria included aphasia, hemineglect, or a history of prior stroke (Barbay et al., 2018). Clinical, neuropsychological, and MRI assessments were performed six months post-injury.

MRI data were acquired using a 3 T scanner (HDXt, General Electric Medical Systems) with an eight-channel head coil. Four sequences were used for lesion segmentation (Arnoux et al., 2018): 3D high-resolution T1-weighted images (T1w; voxel size: 0.8 × 2 × 0.5 mm^3^; TR/TE: 13/4.5 ms), FLAIR (voxel size: 1.16 × 0.68 × 5 mm^3^; TR/TE: 9000/150 ms), T2 (voxel size: 0.609 × 3 × 0.625 mm^3^; TR/TE: 4400/100 ms), and T2*-weighted images (FOV: 195 × 195 × 260 mm; slice thickness: 5 mm; TR/TE: 475/13 ms).

Each patient underwent the full GRECogVASC neuropsychological battery (Table 1), utilizing the French version of the Harmonization Standards battery (Barbay et al., 2018). The results from the clinical tests were assessed using an established analytical framework that incorporated normative cognitive data from a cohort of 1003 healthy individuals (36 % male; mean age ± SD: 62 ± 11.3 years; mean education level ± SD: 11.3 ± 3.1 years, with distribution as follows: 27 % primary school, 37.4 % junior high school, 35.6 % high school; mean Mini-Mental State Examination score: 28.7 ± 1.4; mean Montreal Cognitive Assessment score: 26.5 ± 2.6) (Roussel et al., 2016). Control group cut-off scores were established at the 5th percentile.

**Table 1:** GRECogVASC battery (Barbay et al., 2018).

Domain	Tests	Domain score
Executive functions	Verbal fluency (1 min): animals, letters “P, V, R” Trail Making Test Syndrome Inventory	Z animal, Z ‘PVR’ Z error, part B-part A Total Z score
Processing speed	Trail Making Test Digit Symbol Coding WAIS III	Z time part B Total z score
Motor function	A limb motor score*, corresponding to 10 minus the sum of the upper and lower limb items in the NIHSS	No impairment = 10 Major impairment = 2

*Facial paresis was excluded as it may be caused by a lesion outside of the central motor system.

For deficit prediction, we assessed left/right motor deficits and more complex deficits related to processing speed and executive function. Motor deficits were evaluated using a validated method (Arnoux et al., 2018) based on the National Institute of Health Stroke Scale (NIHSS) (Brott et al., 1989). A limb motor score was derived by subtracting the sum of the upper and lower limb items from 10 (where no impairment = 10 and major impairment = 2), excluding facial paresis, as it may result from lesions outside the central motor system. Cognitive domains, including processing speed and executive function, were evaluated using validated scoring systems previously established by Barbay et al. (2018) and Godefroy et al. (2014). Of the 340 patients assessed, executive function scores were available for 338, while processing speed scores were available for 334 as outlined in Table 2.

**Table 2:** Demographic characteristics of the 340 S patients included in this study.

	Executive Function		Processing Speed		Motor Function		
	Patients (n = 338)		Patients (n = 334)		Patients (n = 340)		
	EFD (n = 118)	No EFD (n = 220)	PSD (n = 137)	No PSD (n = 197)	Left MD (n = 35)	Right MD (n = 32)	No MD (n = 276)
Age (years)	67.08 ± 9.92	62 ± 10.4	66.64 ± 10.02	61.7 ± 10.4	66.5 ± 10.4	62.5 ± 10.4	63.8 ± 10.6
Male (%)	61.86	61.36	60	62	45	56	64
Handedness (Right/Left) (%)	92/8	90/10	90/10	91/9	89 /11	90/10	91/9
Stroke subtype (infarct/hemorrage)(%)	88.9/11.1	92.3/7.7	90/10	92.9/7.1	91.5/8.5	81.2 /18.8	91.6 /8.4
Post-stroke delay (day)	175 ± 20	176 ± 18	176 ± 18	176 ± 18	178 ± 21	174 ± 18	176.4 ± 18.37
NIHSS 6 months	2.7 ± 3.6	0.97 ± 1.69	2.4 ± 3.4	0.85 ± 1.54	6.4 ± 4	4.5 ± 3.11	0.66 ± 0.94
Functional score	−2.8 ± 1.12	−0.61 ± 0.64	−2.7 ± 0.94	−0.48 ± 0.77	Right/Left 10/6.8 ± 2.3	Right/Left 8.2 ± 1.14/10	Right/Left 10/10
Rankin (0/1/2/3/4) (%)	16.1/16.1/ 16.9/38.2/ 12.7	21.4/28.6/ 23.63/22.27/ 4.1	15.3/10.9/6.1/46.8/10.9	22.8/34/ 24.9/14.7/ 3.6	0/ 8.5/ 8.5/ 31.5/51.5	0/ 3.1/ 12.5/62.5/ 21.9	24/28.3/ 23.5/23.5/ 0.7
Lesion volume (mean ± SD, cm^3^)	2.48 ± 3.61	1.06 ± 0.65	2.84 ± 2.77	0.94 ± 0.68	5.84 ± 10.17	1.56 ± 6.24	1.38 ± 0.58

EFD: Executive function deficit; PSD: processing speed deficit; MD: motor deficit.

### 2.2 Normative cohort

For functional and structural connectivity analysis, we used T1w images (voxel size: 1 × 1 × 1 mm^3^), DWI data (voxel size: 2 × 2 × 2 mm^3^), and 8-minute resting-state fMRI (rsfMRI) data (voxel size: 3 × 3 × 4.44 mm^3^, repetition time (TR): 2 s) (see (Taylor et al., 2017) for imaging sequence details). These data were acquired from 403 healthy individuals (mean age 62.87 ± 13.47 years) from the Cam-CAN dataset (Shafto et al., 2014, Taylor et al., 2017), matched to the age range of patients in the GRECogVASC study.

### 2.3 Functional data preprocessing and parcellation

The CamCAN imaging data were preprocessed according to the procedures outlined by Khalilian et al. (2024b). T1-weighted (T1w) images were aligned and normalized to the MNI 152 template, followed by segmentation to delineate brain compartments, including gray matter, white matter, and cerebrospinal fluid (CSF). For resting-state fMRI (rsfMRI) data preprocessing, the initial four volumes were discarded, followed by despiking and slice-timing correction using the middle slice of each volume. Motion correction was performed, with frames exhibiting substantial head motion or rotation (exceeding 3.0 mm translation or 3.0° rotation) being excluded. The rsfMRI data were then realigned and co-registered with the T1w images, spatially normalized to MNI152 space, resliced to 3 mm × 3 mm × 3 mm voxels, and smoothed with a Gaussian kernel (full-width at half-maximum of 6 mm). Mean signals from white matter and CSF were regressed out as temporal covariates using multiple linear regression. Finally, the rsfMRI data were temporally band-pass filtered between 0.01 and 0.1 Hz (Khalilian et al., 2024b).

For graph theoretical analysis, we used the spatially-constrained normalized-cut spectral clustering algorithm for functional parcellation (Craddock et al., 2012). This produced a functional atlas with 1133 functionally homogeneous parcels (regions of interest, ROIs) covering cortical and subcortical regions, deep gray matter, and the cerebellum within the template space (Khalilian et al., 2024b).

### 2.4 Characteristic features

**Fig. 1:**
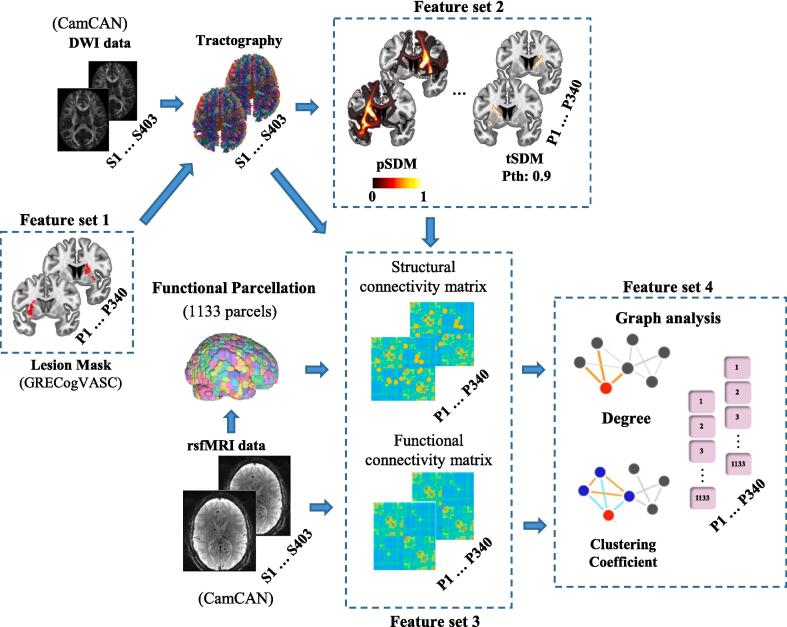
Feature sets utilized for predicting functional deficits in stroke patients using a ridge regression model. A total of 13 features were extracted, including the lesion mask, probabilistic structural disconnection maps (pSDM), their thresholded variants (tSDM) with probability thresholds (Pth) ranging from 0.1 to 0.9, and lesion-derived structural and functional connectivity matrices, along with their nodal degree and clustering coefficients.

As depicted in Fig. 1, four lesion-derived feature sets were extracted and used to predict motor, executive, and processing speed scores in stroke patients.

#### 2.4.1 Feature set 1: Lesion masks

The first feature set comprised whole-brain lesion masks. To derive these masks, imaging data from the GRECogVASC study were preprocessed to delineate lesion regions. Initially, a binary lesion mask was created for each patient by manually segmenting lesions observed on 3D T1w MRI data in native space using MRIcron software, following a previously validated method (Arnoux et al., 2017). FLAIR, T2, and T2* images were used exclusively to assist in lesion identification, ensuring accurate differentiation from the 3D T1w images. Subsequently, the T1w images were normalized to the MNI152 template using SPM12. The resulting lesion segmentations were then converted into binary lesion masks to create structural disconnection maps. Additionally, the volume of each lesion was quantified in the template space for subsequent regression analysis (Khalilian et al., 2024a).

#### 2.4.2 Feature set 2: Lesion-derived structural disconnection maps

Feature set 2 comprised lesion-derived structural disconnection maps for each patient. To generate these maps, the DWI data from 403 healthy subjects were first corrected for susceptibility and eddy current distortions. Deterministic tractography was then performed using the q-space diffeomorphic reconstruction algorithm (Yeh and Tseng, 2011) in MNI space, with angular threshold and step size parameters set to 45° and 0.75 mm, respectively. Streamlines shorter than 30 mm or longer than 300 mm were excluded, followed by topology-informed pruning to remove false connections (Yeh et al., 2019).

For each patient, a whole-brain Probabilistic Structural Disconnection Map (pSDM) was created by integrating the patient’s lesion into the tractography results, as described by Khalilian et al. (2024a). This process involved modifying the deterministic tractography results of each healthy subject by removing white matter streamlines that intersected with the patient's lesion. The pSDM was then constructed across all healthy subjects, with each voxel assigned a disconnection probability ranging from zero to one, indicating the likelihood of disconnection based on the observed frequency across healthy subjects (Khalilian et al., 2024a). The second feature set comprised six probabilistic structural disconnection maps for each patient: one unthresholded pSDM and five thresholded disconnection maps (tSDMs). The tSDMs were created using probability thresholds of 0.1, 0.3, 0.5, 0.7, and 0.9, where the threshold value indicated the minimum proportion of healthy subjects exhibiting disconnection at a given voxel for it to be retained in the map. For instance, a threshold of 0.1 included disconnections observed in at least 10 % of healthy subjects (≥40 out of 403), whereas a threshold of 0.9 retained only those disconnections occurring in at least 90 % of the cohort. This thresholding approach excluded less frequent disconnections, which might otherwise diminish the model's predictive accuracy.

#### 2.4.3 Feature set 3: Lesion-derived weighted connectivity matrices

Feature set 3 comprised lesion-derived structural and functional connectivity matrices. For each patient, a lesion-derived structural connectivity matrix (SC_LD_) was constructed using the functional atlas in MNI space. Initially, a 1133 × 1133 weighted structural connectivity matrix was generated for each healthy subject, with edges representing the number of fibers connecting each pair of ROIs. A group SC matrix (SC_g_) was derived by averaging the weights of edges present in at least 50 % of the healthy subjects (Heuvel and Sporns, 2013). Similarly, a group lesion SC matrix (SC_L_) was obtained after removing streamlines intersecting the patient's lesion from the tractography results of healthy subjects. The SC_LD_ was then calculated by computing the relative changes in connectivity weights between SC_L_ and SC_g_ for each patient.

To generate a lesion-derived functional connectivity matrix (FC_LD_) for each patient, we first constructed a functional connectivity matrix for each healthy subject based on Pearson's correlation coefficients calculated between the mean time courses of ROI pairs. The group functional connectivity matrix (FC_g_) was then computed across all healthy subjects. To simulate the impact of lesions on functional connections, the pSDM of each patient was first thresholded at 0.1 to remove spurious disconnections. Structurally disconnected ROIs were defined as those with a minimum overlap of ten voxels with parcels in the functional atlas. Edges connected to lesion-affected parcels were set to zero in the FC matrix for each subject and averaged across all healthy subjects to obtain an average lesion FC matrix (FC_L_). The lesion-derived FC matrix was then computed by computing the relative change in connectivity weights between FC_L_ and FC_g_ for each patient.

Out of 340 subjects, 11 were excluded from the functional analysis because their lesions were too small to meet the minimum overlap criterion of ten voxels with parcels in the functional atlas.

Both structural and functional procedures were conducted for each patient, yielding a total of 340 matrices for SC_LD_ and 329 matrices for FC_LD_. Elements from the upper triangles of these matrices were included in Feature set 3 for predicting functional deficits.

#### 2.4.4 Feature set 4: Lesion-derived connectivity measures

To assess the impact of lesions on the local topological characteristics of both structural and functional brain networks, we analyzed the relative changes in nodal parameters − specifically strength (K) and clustering coefficient (Cc) − within the lesion structural/functional connectivity (SC_L_/FC_L_) matrices, compared to the corresponding intact graphs (SCg/FCg) for each patient. In weighted networks, the strength of a node signifies the sum of edge weights connected to it, reflecting its level of connectivity within the network. Conversely, the clustering coefficient quantifies the extent to which the neighbors of a node are interconnected, offering insights into the local density of connections around that node, considering the weights of the edges. For each patient, Feature set 4 comprised four vectors of 1133 elements, representing the relative lesion-derived changes in either K or C_C_ of the nodes within the structural/functional networks.

### 2.5 Feature dimensionality reduction

A total of 13 feature vectors were used for regression analysis per patient. Lesion volumes, obtained from lesion masks, were also included in the prediction process. Feature sets 1 and 2 consisted of feature vectors with over 1.3 million and 500,000 elements, respectively, corresponding to the number of voxels in whole-brain lesion masks and probabilistic structural disconnection maps. Feature set 3 included two feature vectors per patient, each containing over 600,000 elements derived from the upper triangle of the SC_LD_ or FC_LD_ matrices. Feature set 4 comprised four feature vectors per patient, each with 1,133 elements corresponding to network nodes.

To predict each deficit, we constructed a P × N feature matrix for each feature, where P represents the number of patients and N denotes the number of elements in the feature vector for each patient. Given the high dimensionality of the feature space, which could impact prediction performance, we first eliminated zero-value elements from the feature vectors across all patients. Then, for each feature matrix, we performed PCA on the entire dataset to reduce dimensionality, retaining PCs that explained 99 % of the variance.

### 2.6 Prediction model and performance evaluation

We employed Ridge regression due to its resilience to sample size variations in lesion-behavior mapping, as compared to alternative methods (Chauhan et al., 2019). Unlike multiple linear regression, the Ridge regression uses L2-normalization to regularize model coefficients, reducing the influence of less significant features and thus preventing overfitting and enhancing model generalization on test data (Le Cessie and Van Houwelingen, 1992).

To predict functional deficits at the individual level, a leave-one-out cross-validation (LOOCV) strategy was utilized for prediction model optimization and evaluation. Coefficients of determination (R^2^ values) were calculated to compare predicted and actual scores in motor function, executive function, and processing speed (Corbetta et al., 2015, Golland and Fischl, 2003, Salvalaggio et al., 2020, Siegel et al., 2016).

**Training strategy for regression model optimization**: A two-step nested training algorithm with LOOCV, similar to the approach introduced by Varma and Simon (2006), was employed to optimize the regression model's performance for each feature and functional score. For every test patient, a training set was first constructed using PCs associated with the feature from other patients. These PCs were then ranked in ascending order based on their correlation with the functional scores of the training patients. This process was iterated for all test patients, and the aggregated PC rankings from all training sets were used to establish a unique PC ranking order for each feature. Subsequently, LOOCV was applied to all training sets to identify the average optimal number of ranked PCs that maximized R^2^ across the validation patients within the training sets. Additionally, the optimal regularization coefficient (λ) was selected from the range [0.001, 100] using logarithmic steps to maximize R^2^ on the validation patients. This approach minimized LOOCV prediction error while preventing overfitting and underfitting (Salvalaggio et al., 2020).

**Model testing:** The optimal PC ranking order and number of PCs for each feature and functional score were used to rearrange the PCs and evaluate prediction performance on test patients using LOOCV (code available on https://github.com/Maedehkh/Post-Stroke-Deficit-Prediction).

Performance metrics, including R^2^, mean squared error (MSE), and total explained variance, were reported for each model. The statistical significance of the LOOCV results was evaluated using permutation testing. In this approach, functional scores were randomized across participants 10,000 times for each optimal PC set and corresponding score, and Ridge regression models were fitted to these permuted datasets. The p-value was determined by calculating the proportion of permutations that produced R^2^ values exceeding the observed R^2^. Models yielding p-values below 0.05 were considered statistically significant.

For each deficit, we performed a statistical comparison between the regression model that achieved the highest R2 using the most effective feature against the others by using model confidence intervals via Fisher's z-transformation. This approach enables a more robust comparison of R2 values, particularly when correlation coefficient distributions may be skewed. First, R2 values were transformed to z-scores using Fisher's z-transformation. The standard error of the z-scores was then calculated to construct their confidence intervals. These intervals were converted back to the R2 scale using the inverse Fisher transformation. Finally, we compared the confidence intervals to determine if the differences in R2 values between the top-performing model and the others were statistically significant (Lawson et al., 2024, Loughnan et al., 2019).

## 3 Results

**Fig. 2:**
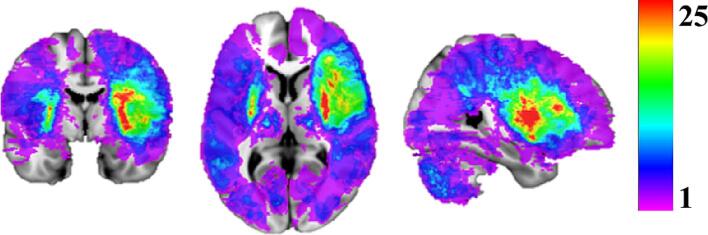
Lesion overlap maps for 340 S patients. Colorbars show the number of patients with overlapping lesions at each voxel. Maps are overlaid on the MNI 152 template in MRIcron.

Fig. 2 presents the lesion overlap map for all 340 S patients, with the mean lesion volume (± SD) for each deficit summarized in Table 2.

**Fig. 3:**
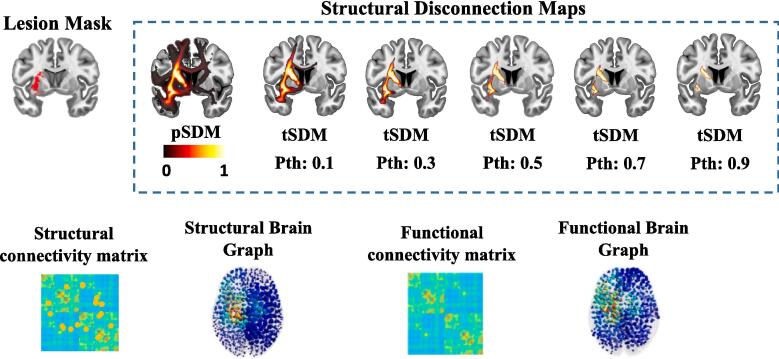
Features extracted from the lesion mask of Patient 1 include the lesion mask, a probabilistic structural disconnection map (pSDM) with thresholded variants (tSDM) at probability thresholds ranging from 0.1 to 0.9, and lesion-derived structural and functional connectivity matrices, along with the associated graphs. In the connectivity matrices and graphs, the color of elements and nodes represents connection strength and nodal degree, with lower values depicted in blue and higher values in yellow. (For interpretation of the references to color in this figure legend, the reader is referred to the web version of this article.)

Fig. 3 presents the features extracted from Patient 1′s lesion mask, including the lesion mask itself, the probabilistic structural disconnection maps (pSDM) and their thresholded variants (tSDM) across probability thresholds ranging from 0.1 to 0.9, along with the lesion-derived structural and functional connectivity matrices and their respective graph representations.

Table 3 presents the regression results for predicting motor, executive function, and processing speed scores using each feature with the leave-one-out cross-validation (LOOCV) approach, employing λ = 0.01 as the optimal value consistently applied across all prediction models. Lesion volume proved to be a poor predictor, with the highest R^2^ value falling below 0.22 for left motor deficits in LOOCV. In contrast, SDM patterns, lesion masks, and modifications in structural connection strengths were significantly more predictive of functional deficits compared to structural and functional nodal parameters. In particular, lesion masks exhibited greater predictive efficacy than unthresholded pSDMs. Moreover, excluding disconnections with lower probabilities improved prediction accuracy.

**Table 3:** Regression results for predicting motor, executive function and processing speed deficits using the LOOCV strategy.

				R^2^		MSE	# of PCs	TEV (%)	p-value (Fisher *r*-to-*z* transform)
Left motor function	Lesion volume			0.22	p < 10^-8^	0.88	−	−	0.000
	Lesion masks			0.86	p < 10^-8^	0.13	97	70	0.000
	**pSDM**	No threshold		0.82	p < 10^-8^	0.17	105	70	0.000
	**tSDM**	P_th_ = 0.1		0.89	p < 10^-8^	0.10	151	91	0.000
		P_th_ = 0.3		0.92	p < 10^-8^	0.07	167	86	0.006
		P_th_ = 0.5		0.92	p < 10^-8^	0.07	141	84	0.006
		**P_th_ = 0.7**		**0.94**	**p < 10^-8^**	**0.05**	**143**	**83**	**1.00**
		**P_th_ = 0.9**		**0.94**	**p < 10^-8^**	**0.05**	**167**	**91**	**1.00**
	**Lesion-derived changes**	SN	CW	0.91	p < 10^-8^	0.08	153	89	0.000
			K	0.74	p < 10^-8^	0.25	49	49	0.000
			C_C_	0.83	p < 10^-8^	0.16	45	92	0.000
		FN	CW	0.34	p < 10^-8^	0.65	11	75	0.000
			K	0.18	p < 10^-8^	0.81	5	4	0.000
			C_C_	0.81	p < 10^-8^	0.17	61	89	0.000
Right motor function	Lesion volume			−6e-4	p = 0.9	0.99	−	−	0.000
	Lesion masks			0.46	p < 10^-2^	0.53	77	57	0.000
	**pSDM**	No threshold		0.44	p < 10^-2^	0.55	95	70	0.000
	**tSDM**	P_th_ = 0.1		0.43	p < 0.05	0.56	97	66	0.000
		**P_th_ = 0.3**		**0.65**	p < 10^-4^	**0.34**	**137**	**80**	**0.17**
		**P_th_ = 0.5**		**0.69**	p < 10^-8^	**0.30**	**125**	**75**	**1.00**
		P_th_ = 0.7		0.61	p < 10^-2^	0.38	131	69	0.009
		P_th_ = 0.9		0.63	p < 10^-8^	0.36	105	67	0.046
	**Lesion-derived changes**	SN	CW	0.49	p < 10^-2^	0.50	111	63	0.000
			K	0.26	p < 10^-2^	0.73	37	60	0.000
			C_C_	0.23	p < 10^-2^	0.76	25	79	0.000
		FN	CW	0.05	p < 10^-2^	0.94	1	0.4	0.000
			K	0.02	P = 0.2	0.97	5	94	0.000
			Cc	0.29	P = 0.07	0.70	59	87	0.000
Executive function	Lesion volume			0.04	p < 10^-4^	0.97	−	−	0.000
	Lesion masks			0.45	p < 10^-2^	0.54	109	67	0.004
	pSDM	No threshold		0.27	P = 0.16	0.72	81	71	0.000
	tSDM	P_th_ = 0.1		0.46	p < 10^-8^	0.53	85	66	0.009
		**P_th_ = 0.3**		**0.56**	p < 10^-8^	**0.43**	**117**	**71**	**1.00**
		**P_th_ = 0.5**		**0.5**	p < 10^-8^	**0.49**	**103**	**64**	**0.167**
		**P_th_ = 0.7**		**0.48**	p < 0.05	**0.51**	**135**	**78**	**0.056**
		**P_th_ = 0.9**		**0.51**	p < 0.05	**0.48**	**143**	**82**	**0.177**
	Lesion-derived changes	SN	**CW**	**0.51**	p < 10^-8^	**0.48**	**111**	**67**	**0.178**
			K	0.22	p < 10^-3^	0.76	41	72	0.000
			C_C_	0.22	p < 10^-8^	0.77	35	81	0.000
		FN	CW	0.03	p < 0.05	0.95	3	0.76	0.000
			K	0.02	P = 0.14	0.97	5	95	0.000
			C_C_	0.26	p < 10^-2^	0.73	49	80	0.000
Processing speed	Lesion volume			0.05	p < 10^-8^	0.97	−	−	0.000
	Lesion masks			0.54	p < 10^-8^	0.45	117	68	0.023
	pSDM	No threshold		0.33	p < 10^-8^	0.66	43	57	0.000
	tSDM	P_th_ = 0.1		0.49	p < 10^-8^	0.50	99	72	0.000
		**P_th_ = 0.3**		**0.58**	p < 10^-8^	**0.41**	**133**	**72**	**0.24**
		**P_th_ = 0.5**		**0.62**	p < 10^-8^	**0.37**	**139**	**78**	**1.00**
		P_th_ = 0.7		0.55	p < 10^-8^	0.43	125	76	0.044
		P_th_ = 0.9		0.55	p < 10^-3^	0.43	135	78	0.044
	Lesion-derived changes	SN	CW	0.51	p < 10^-8^	0.48	95	69	0.002
			K	0.32	p < 10^-8^	0.66	37	65	0.000
			C_C_	0.25	p < 10^-8^	0.74	39	85	0.000
		FN	CW	0.12	p < 10^-8^	0.86	7	46	0.000
			K	0.03	P < 0.05	0.96	5	96	0.000
			C_C_	0.26	p < 10^-3^	0.73	35	85	0.000

RMSE: root mean square error, ρ: correlation coefficient between real and predicted scores, pSDM: probabilistic structural disconnection map, tSDM: thresholded SDM, PC: Principle component, EV: explained variance, P_th_: probability threshold, CW: connection weight, SN: structural network, FN: functional network, K: degree, Cc: clustering coefficient. TEV: Total explained variance by the optimal PC set. The most effective feature for each deficit is highlighted in blue. The last column presents the p-values obtained using the Fisher r-to-z transformation, which compares the most effective features to other features for each deficit.

**Fig. 4:**
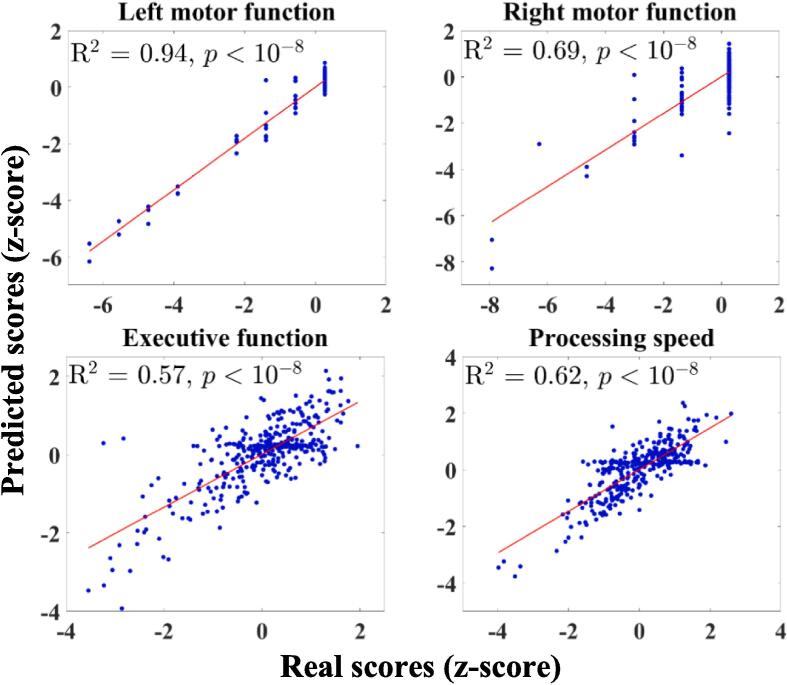
Scatter plots and linear fits depict the coefficient of determination (R^2^) between predicted functional scores and actual scores, acquired through leave-one-out cross-validation. The highest R^2^ values for left and right motor deficits, executive function, and processing speed were obtained using the structural disconnection maps thresholded at probabilities of 0.9, 0.5, 0.3, and 0.5, respectively.

For motor deficits, the regression analysis using thresholded SDMs at probabilities of 0.9 and 0.5 produced the highest R2 values of 0.94 and 0.69 for left and right motor deficits, respectively (see Table 3 and Fig. 4). However, subsequent statistical testing with Fisher's transformation indicated no significant difference between the R2 values obtained using SDMs thresholded at 0.7 versus 0.9 for left motor deficits, and at 0.3 versus 0.5 for right motor deficits. The superior motor performance observed for left motor deficits compared to right motor deficits may stem from the greater spatial overlap of right-side lesions in our dataset. This overlap allowed PCA to better capture variance in lesion/disconnection maps for left motor deficits, enhancing their representativeness of the lesion space and improving the prediction model's performance.

For more complex tasks involving executive function and processing speed, the R^2^ values obtained using the SDM features did not exceed 0.62. As illustrated in Fig. 4, tSDMs produced the highest R^2^ values of 0.56 for executive function and 0.62 for processing speed, with probability thresholds set at 0.3 and 0.5, respectively. Increasing the threshold further did not enhance the R^2^ values for executive function deficits. Additionally, no significant differences were found between the regression results using tSDMs with thresholds of 0.3 and 0.5 for processing speed.

The connectome-based features exhibited lower R^2^ values compared to the other feature types. Among the parameters derived from the structural connectome, lesion-derived alterations in structural connection strength showed a stronger association with functional scores, whereas changes in functional connection strength had minimal correlations with deficits. Nodal parameters generally demonstrated lower explanatory power compared to SDMs. Specifically, the clustering coefficient achieved higher R^2^ values than degree for motor scores, although these values were still significantly lower than those for structural connectivity metrics. Conversely, for executive function and processing speed, degree exhibited slightly higher predictive power than the clustering coefficient. The number of PCs that maximized R^2^ varied depending on the complexity of the input feature space. Interestingly, PCs that exhibited higher correlations with functional scores and achieved higher R^2^ values were not necessarily those with higher explanatory power (eigen values).

## 4 Discussion

In this study, we assessed the efficacy of features derived from structural disconnection maps and lesion-derived topological properties of both structural and functional networks in predicting post-stroke deficits in motor function, executive function, and processing speed. We employed a dysconnectome approach that used simulated connectivity based on data from healthy subjects, rather than relying on direct measurements from stroke patients. This method effectively addresses the challenge of obtaining high-quality DWI and fMRI data from the patient population (Rivier et al., 2023, Salvalaggio et al., 2020).

Our results demonstrated that both SDM patterns and lesion masks were highly predictive of functional deficits. Lesion masks showed higher predictive performance compared to unthresholded pSDMs across all functional scores (see Table 3). This discrepancy may be attributable to unthresholded pSDMs reflecting methodological artifacts rather than actual changes in structural connectivity (Salvalaggio et al., 2020). The SDMs at a probability of 0.1 showed comparable performance to lesion masks in explaining variability in functional scores. This finding is consistent with Salvalaggio et al. (2020), who reported no significant difference between lesion masks and SDMs across various functional domains.

We further evaluated the impact of varying pSDM probability thresholds on prediction performance. Previous studies have investigated different thresholds to assess analysis reliability (Pan et al., 2022, Souter et al., 2022). Our findings indicate that probability thresholds higher than 0.1 were associated with improved prediction accuracy. This observation aligns with the recommendation of Wawrzyniak et al. (2022), who suggested using higher thresholds for tSDM maps to enhance sensitivity and specificity to behavioral scores.

However, our analysis also revealed that extremely high probability thresholds do not necessarily enhance prediction accuracy beyond the levels achieved with lesion masks. For motor deficits, threshold ranges of [0.7 0.9] and [0.3 0.5] for tSDMs resulted in the highest R^2^ values for left and right motor deficits, respectively. The superior motor performance for left motor deficits compared to right motor deficits may result from the higher spatial overlap of right-side lesions in our dataset (Fig. 2), enabling PCA to more effectively capture the variance in lesion/disconnection maps for left motor deficits, making them more representative of the lesion space. Consequently, this could significantly enhance the prediction model's performance for left motor deficits.

In contrast, for more complex tasks (executive function and processing speed), the highest R^2^ values were achieved with tSDM thresholds within [0.3 0.9] and [0.3 0.5], respectively. This finding aligns with Zhao et al. (2022), who reported variability in the predictive accuracy of connectivity measurements across functional domains. Neuroanatomically, the domains explored in this study depend on distinct neural networks. Motor performance depends heavily on focal pathways like the corticospinal tract and motor-related regions, such as the primary motor cortex and supplementary motor area (Byblow et al., 2015, Cho et al., 2007, Godefroy et al., 1998, Kassubek et al., 2005, Lindenberg et al., 2010, Zhu et al., 2010). In contrast, executive function relies on distributed prefrontal-subcortical networks encompassing the dorsolateral prefrontal cortex, anterior cingulate cortex, and their interactions with subcortical structures (Bettcher et al., 2016, Diamond, 2013, Roussel et al., 2016). Processing speed depends on the global efficiency of white matter tracts, such as the superior longitudinal fasciculus, which facilitates inter-network information transfer (Duering et al., 2011, Godefroy et al., 2024, Ouin et al., 2022). The reliance of these domains on distributed connectivity likely accounts for their respective predictive accuracies when assessed with the neuroimaging features examined in this study.

The connectome-based features exhibited lower R^2^ values compared to other features. Specifically, among the structural connectome parameters, changes in structural connection strength derived from lesions showed a stronger association with post-stroke outcomes, comparable to lesion masks. This finding is consistent with Griffis et al. (2020), which emphasized the significance of structural connectivity in predicting functional outcomes. Conversely, we observed minimal correlation between changes in functional connection strength and deficits. This contrasts with the findings of Salvalaggio et al. (2020), who demonstrated that post-stroke alterations in functional connectivity could predict outcomes similarly to lesion-based assessments, potentially due to the use of real patient data, which may improve prediction accuracy. This discrepancy highlights the limitations associated with simulated functional connectivity data.

Nodal parameters in both structural and functional networks generally provided lower explanatory power compared to SDM features and lesion masks. This finding contrasts with Siegel et al. (2016), which identified graph theoretical measures as highly predictive of functional outcomes. However, our connectome-based features, derived from simulated data, may not fully capture the complexity of real patient networks. Rivier et al. (2023) demonstrated that incorporating simulated structural connectivity from healthy subjects improved prediction accuracy (R^2^ = 0.68) compared to using benchmark features alone (R^2^ = 0.38). This suggests that while connectivity measures alone may not outperform other methods, they can enhance prediction accuracy when used in conjunction with additional features. In contrast, Hope et al. (2018) found that lesion location was the strongest predictor of language scores, with structural connectivity contributing only modest additional explanatory power. Similarly, Halai et al. (2020) reported that although structural connectivity played a role in predicting aphasia, it did not surpass the predictive power of lesion-based models. Zhao et al. (2022) confirmed that connectivity measures were predictive of cognitive outcomes but did not account for substantial additional variance beyond lesion characteristics. These findings suggest that graph-theoretical metrics, which simplify brain network properties, are valuable for detecting global changes in neurological conditions like stroke and monitoring treatment responses (Bonilha et al., 2016). However, these metrics do not offer detailed anatomical information, limiting their ability to pinpoint specific brain regions or tracts involved in behavioral deficits or pathophysiological changes (Sperber et al., 2022). The variability in findings across studies underscores the challenges of integrating connectivity measures into predictive models. These discrepancies often stem from differences in methodology, including lesion delineation techniques, feature extraction approaches, and validation strategies. Studies using real patient-derived functional connectivity data typically yield higher predictive accuracy than those based on simulated networks (Salvalaggio et al., 2020). Our findings indicate that although connectivity measures offer useful additional insights, lesion masks and structural network models are the most reliable predictors of post-stroke deficits. To enhance the understanding of post-stroke outcomes, future research should focus on refining connectivity modeling techniques and investigating their interactions with lesion-based features.

### 4.1 Limitations

The current study has several limitations. Firstly, PCA might oversimply the data, potentially losing valuable variance relevant for behavioral prediction (Cohen et al., 2021). Secondly, we used LOOCV to predict individual functional outcomes. This method is particularly useful for imbalanced datasets where the number of patients with deficits is much smaller than those without, as it provides an unbiased estimate of model performance for each subject. However, k-fold cross-validation, which splits the dataset into multiple folds, can offer a more stable and realistic estimate of model performance by reducing variance and assessing generalizability. That said, k-fold cross-validation works best with more balanced datasets, as it may not adequately represent minority patient groups in cases of significant imbalance. Additionally, our functional connectivity analysis was based on structural disconnection patterns, which might not account for changes in indirect functional connectivity. Finally, predicting real-world scores requires longitudinal data, but our study used scores from a single time point (6 months post-stroke) to compare different measurement efficacy. Future research needs to address these limitations to enhance the accuracy and applicability of findings.

## 5 Conclusion

Our study demonstrates that lesion masks and thresholded structural disconnection maps are highly effective in predicting post-stroke deficits in motor function, executive function, and processing speed. Lesion masks generally outperformed unthresholded SDMs. However, appropriately thresholded SDMs showed higher predictive power than lesion masks, highlighting the importance of optimal threshold selection to enhance model accuracy. Simulated structural connectivity showed comparable performance to lesion masks, whereas simulated functional connectivity proved significantly less effective. Nodal parameters for both functional and structural networks exhibited lower predictive capability compared to lesion masks and SDM patterns. Overall, our findings underscore the value of appropriately thresholded SDMs and contribute to the growing evidence supporting the reliability of simulated structural networks as a complementary approach to lesion patterns and structural disconnections in predicting post-stroke outcomes.

## CRediT authorship contribution statement

**Maedeh Khalilian:** Writing – original draft, Methodology, Formal analysis, Data curation, Conceptualization. **Olivier Godefroy:** Writing – review & editing, Investigation, Funding acquisition, Conceptualization. **Martine Roussel:** Writing – review & editing. **Amir Mousavi:** Writing – review & editing. **Ardalan Aarabi:** Writing – review & editing, Supervision, Methodology, Investigation, Conceptualization.

## Declaration of Competing Interest

The authors declare that they have no known competing financial interests or personal relationships that could have appeared to influence the work reported in this paper.

## Data Availability

The authors do not have permission to share data.

## References

[b0005] Arnoux A., Toba M.N., Duering M., Diouf M., Daouk J., Constans J.-M., Puy L., Barbay M., Godefroy O. (2018). Is VLSM a valid tool for determining the functional anatomy of the brain? Usefulness of additional Bayesian network analysis. Neuropsychologia.

[b0010] Arnoux A., Triquenot-Bagan A., Andriuta D., Wallon D., Guegan-Massardier E., Leclercq C., Martinaud O., Castier-Amouyel M., Godefroy O., Bugnicourt J.-M. (2017). Imaging Characteristics of Venous Parenchymal Abnormalities. Stroke.

[b0015] Barbay M., Taillia H., Nédélec-Ciceri C., Bompaire F., Bonnin C., Varvat J., Grangette F., Diouf M., Wiener E., Mas J.-L., Roussel M., Godefroy O. (2018). Prevalence of Poststroke Neurocognitive Disorders Using National Institute of Neurological Disorders and Stroke-Canadian Stroke Network, VASCOG Criteria (Vascular Behavioral and Cognitive Disorders), and Optimized Criteria of Cognitive Deficit. Stroke.

[b0020] Bates E., Wilson S.M., Saygin A.P., Dick F., Sereno M.I., Knight R.T., Dronkers N.F. (2003). Voxel-based lesion-symptom mapping. Nat. Neurosci..

[b0025] Bettcher B.M., Mungas D., Patel N., Elofson J., Dutt S., Wynn M., Watson C.L., Stephens M., Walsh C.M., Kramer J.H. (2016). Neuroanatomical Substrates of Executive Functions: Beyond Prefrontal Structures. Neuropsychologia.

[b0030] Boes A.D., Prasad S., Liu H., Liu Q., Pascual-Leone A., Caviness V.S., Fox M.D. (2015). Network localization of neurological symptoms from focal brain lesions. Brain.

[b0035] Bonilha L., Gleichgerrcht E., Nesland T., Rorden C., Fridriksson J. (2016). Success of Anomia Treatment in Aphasia Is Associated With Preserved Architecture of Global and Left Temporal Lobe Structural Networks. Neurorehabil. Neural Repair.

[b0040] Boyd L.A., Hayward K.S., Ward N.S., Stinear C.M., Rosso C., Fisher R.J., Carter A.R., Leff A.P., Copland D.A., Carey L.M., Cohen L.G., Basso D.M., Maguire J.M., Cramer S.C. (2017). Biomarkers of stroke recovery: Consensus-based core recommendations from the Stroke Recovery and Rehabilitation Roundtable. Int. J. Stroke off. J. Int. Stroke Soc..

[b0045] Brott T., Adams H.P., Olinger C.P., Marler J.R., Barsan W.G., Biller J., Spilker J., Holleran R., Eberle R., Hertzberg V. (1989). Measurements of acute cerebral infarction: a clinical examination scale. Stroke.

[b0050] Byblow W.D., Stinear C.M., Barber P.A., Petoe M.A., Ackerley S.J. (2015). Proportional recovery after stroke depends on corticomotor integrity. Ann. Neurol..

[b0055] Carter A.R., Astafiev S.V., Lang C.E., Connor L.T., Rengachary J., Strube M.J., Pope D.L.W., Shulman G.L., Corbetta M. (2010). Resting interhemispheric functional magnetic resonance imaging connectivity predicts performance after stroke. Ann. Neurol..

[b0060] Chauhan S., Vig L., Grazia D.F.D., M., Corbetta, M., Ahmad, S., Zorzi, M., (2019). A Comparison of Shallow and Deep Learning Methods for Predicting Cognitive Performance of Stroke Patients From MRI Lesion Images. Front. Neuroinformatics.

[b0065] Cho S.-H., Kim D.G., Kim D.-S., Kim Y.-H., Lee C.-H., Jang S.H. (2007). Motor outcome according to the integrity of the corticospinal tract determined by diffusion tensor tractography in the early stage of corona radiata infarct. Neurosci. Lett..

[b0070] Cohen A.L., Ferguson M.A., Fox M.D. (2021). Lesion network mapping predicts post-stroke behavioural deficits and improves localization. Brain.

[b0075] Corbetta, D., Sirtori, V., Castellini, G., Moja, L., Gatti, R., 2015. Constraint-induced movement therapy for upper extremities in people with stroke. Cochrane Database Syst. Rev. 2015, CD004433. 10.1002/14651858.CD004433.pub3. 10.1002/14651858.CD004433.pub3 PMC6465192 26446577

[b0080] Corbetta M., Kincade M.J., Lewis C., Snyder A.Z., Sapir A. (2005). Neural basis and recovery of spatial attention deficits in spatial neglect. Nat. Neurosci..

[b0085] Craddock R.C., James G.A., Holtzheimer P.E., Hu X.P., Mayberg H.S. (2012). A whole brain fMRI atlas generated via spatially constrained spectral clustering. Hum. Brain Mapp..

[b0090] Diamond A. (2013). Executive Functions. Annu. Rev. Psychol..

[b0095] Duering M., Zieren N., Hervé D., Jouvent E., Reyes S., Peters N., Pachai C., Opherk C., Chabriat H., Dichgans M. (2011). Strategic role of frontal white matter tracts in vascular cognitive impairment: a voxel-based lesion-symptom mapping study in CADASIL. Brain.

[b0100] Ferris J., Greeley B., Yeganeh N.M., Rinat S., Ramirez J., Black S., Boyd L. (2022). Exploring biomarkers of processing speed and executive function: The role of the anterior thalamic radiations. NeuroImage Clin..

[b0105] Foulon C., Cerliani L., Kinkingnéhun S., Levy R., Rosso C., Urbanski M., Volle E., Thiebaut de Schotten M. (2018). Advanced lesion symptom mapping analyses and implementation as BCBtoolkit. GigaScience.

[b0110] Fox M.D. (2018). Mapping Symptoms to Brain Networks with the Human Connectome. N. Engl. J. Med..

[b0115] Godefroy O., Duhamel A., Leclerc X., Saint Michel T., Hénon H., Leys D. (1998). Brain-behaviour relationships. Some models and related statistical procedures for the study of brain-damaged patients. Brain. J. Neurol..

[b0120] Godefroy O., Gibbons L., Diouf M., Nyenhuis D., Roussel M., Black S., Bugnicourt J.M. (2014). Validation of an integrated method for determining cognitive ability: Implications for routine assessments and clinical trials. Cortex.

[b0125] Godefroy O., Leclercq C., Roussel M., Moroni C., Quaglino V., Beaunieux H., Tallia H., Nédélec-Ciceri C., Bonnin C., Thomas-Anterion C., Varvat J., Aboulafia-Brakha T., Assal F. (2012). French Adaptation of the Vascular Cognitive Impairment Harmonization Standards: The GRECOG-VASC Study. Int. J. Stroke.

[b0130] Godefroy O., Weaver N.A., Roussel M., Dorchies F., Kassir R., Biesbroek J.M., Lee K.-J., Kim B.J., Bae H.-J., Lim J.-S., Lee M., Yu K.-H., Aben H.P., de Kort P.L.M., Bordet R., Lopes R., Dondaine T., Biessels G.J., Aarabi A., the MetaVCI map consortium, (2024). Architecture and anatomy of executive processes: evidence from verbal fluency and Trail Making Test in 2009 stroke patients. J. Neurol..

[b0135] P. Golland B. Fischl Permutation Tests for Classification: Towards Statistical Significance in Image-Based Studies C. Taylor J.A. Noble Information Processing in Medical Imaging Lecture Notes in Computer Science 2003 Springer Berlin, Heidelberg 330 341 10.1007/978-3-540-45087-0_28. 10.1007/978-3-540-45087-0_28 15344469

[b0140] Griffis J.C., Metcalf N.V., Corbetta M., Shulman G.L. (2020). Damage to the shortest structural paths between brain regions is associated with disruptions of resting-state functional connectivity after stroke. NeuroImage.

[b0145] Halai A.D., Woollams A.M., Lambon Ralph M.A. (2020). Investigating the effect of changing parameters when building prediction models for post-stroke aphasia. Nat. Hum. Behav..

[b0150] van den Heuvel M.P., Sporns O. (2013). An Anatomical Substrate for Integration among Functional Networks in Human Cortex. J. Neurosci..

[b0155] Hope T.M.H., Leff A.P., Price C.J. (2018). Predicting language outcomes after stroke: Is structural disconnection a useful predictor?. NeuroImage Clin..

[b0160] Ivanova M.V., Herron T.J., Dronkers N.F., Baldo J.V. (2021). An empirical comparison of univariate versus multivariate methods for the analysis of brain–behavior mapping. Hum. Brain Mapp..

[b0165] Kassubek J., Unrath A., Huppertz H.-J., Lulé D., Ethofer T., Sperfeld A.-D., Ludolph A.C. (2005). Global brain atrophy and corticospinal tract alterations in ALS, as investigated by voxel-based morphometry of 3-D MRI. Amyotroph. Lateral Scler. Mot. Neuron Disord. Off. Publ. World Fed. Neurol. Res. Group Mot. Neuron Dis..

[b0170] Khalilian M., Roussel M., Godefroy O., Aarabi A. (2024). Predicting functional impairments with lesion-derived disconnectome mapping: Validation in stroke patients with motor deficits. Eur. J. Neurosci..

[b0175] Khalilian M., Toba M.N., Roussel M., Tasseel-Ponche S., Godefroy O., Aarabi A. (2024). Age-related differences in structural and resting-state functional brain network organization across the adult lifespan: A cross-sectional study. Aging Brain.

[b0180] Kuceyeski A., Navi B.B., Kamel H., Raj A., Relkin N., Toglia J., Iadecola C., O’Dell M. (2016). Structural connectome disruption at baseline predicts 6-months post-stroke outcome. Hum. Brain Mapp..

[b0185] Lawson B., Martin J., Aarabi A., Ouin E., Tasseel-Ponche S., Barbay M., Andriuta D., Roussel M., Godefroy O., Godefroy O., Roussel M., Barbay M., Canaple S., Lamy C., Leclercq C., Arnoux A., Despretz-Wannepain S., Despretz P., Berrissoul H., Picard C., Diouf M., Loas G., Deramond H., Taillia H., Ardisson A.-E., Nédélec-Ciceri C., Bonnin C., Thomas-Anterion C., Vincent-Grangette F., Varvat J., Quaglino V., Beaunieux H., Moroni C., Martens-Chazelles A., Batier-Monperrus S., Monteleone C., Costantino V., Theunssens E. (2024). Poststroke cognitive outcome is better accounted for by white matter abnormalities automated segmentation than visual analysis. Rev. Neurol. (paris)..

[b0190] Le Cessie S., Van Houwelingen J.C. (1992). Ridge Estimators in Logistic Regression. J. R. Stat. Soc. Ser. C Appl. Stat..

[b0195] Liang X., Koh C.-L., Yeh C.-H., Goodin P., Lamp G., Connelly A., Carey L.M. (2021). Predicting Post-Stroke Somatosensory Function from Resting-State Functional Connectivity: A Feasibility Study. Brain Sci..

[b0200] Lindenberg R., Renga V., Zhu L.L., Betzler F., Alsop D., Schlaug G. (2010). Structural integrity of corticospinal motor fibers predicts motor impairment in chronic stroke. Neurology.

[b0205] Loughnan R., Lorca-Puls D.L., Gajardo-Vidal A., Espejo-Videla V., Gillebert C.R., Mantini D., Price C.J., Hope T.M.H. (2019). Generalizing post-stroke prognoses from research data to clinical data. NeuroImage Clin..

[b0210] Ouin E., Roussel M., Aarabi A., Arnoux A., Tasseel-Ponche S., Andriuta D., Thiebaut de Schotten M., Toba M.N., Makki M., Godefroy O. (2022). Poststroke action slowing: Motor and attentional impairments and their imaging determinants. Evidence from lesion-symptom mapping, disconnection and fMRI activation studies. Neuropsychologia.

[b0215] Pan C., Li G., Jing P., Chen G., Sun W., Miao J., Wang Y., Lan Y., Qiu X., Zhao X., Mei J., Huang S., Lian L., Wang H., Zhu Z., Zhu S. (2022). Structural disconnection-based prediction of poststroke depression. Transl. Psychiatry.

[b0220] Puig J., Blasco G., Alberich-Bayarri A., Schlaug G., Deco G., Biarnes C., Navas-Martí M., Rivero M., Gich J., Figueras J., Torres C., Daunis-i-Estadella P., Oramas-Requejo C.L., Serena J., Stinear C.M., Kuceyeski A., Soriano-Mas C., Thomalla G., Essig M., Figley C.R., Menon B., Demchuk A., Nael K., Wintermark M., Liebeskind D.S., Pedraza S. (2018). Resting-State Functional Connectivity Magnetic Resonance Imaging and Outcome After Acute Stroke. Stroke.

[b0225] Puy, L., Barbay, M., Roussel, M., Canaple, S., Lamy, C., Arnoux, A., Leclercq, C., Mas, J.-L., Tasseel-Ponche, S., Constans, J.-M., Godefroy, O., GRECogVASC Study Group (2018). Neuroimaging Determinants of Poststroke Cognitive Performance. Stroke.

[b0230] Rivier C., Preti M.G., Nicolo P., Van De Ville D., Guggisberg A.G., Pirondini E. (2023). Prediction of post-stroke motor recovery benefits from measures of sub-acute widespread network damages. Brain Commun..

[b0235] Roussel M., Martinaud O., Hénon H., Vercelletto M., Bindschadler C., Joseph P.-A., Robert P., Labauge P., Godefroy O. (2016). The Behavioral and Cognitive Executive Disorders of Stroke: The GREFEX Study. PLoS ONE.

[b0240] Salvalaggio A., Grazia D.F.D., M., Zorzi, M., Thiebaut de Schotten, M., Corbetta, M., (2020). Post-stroke deficit prediction from lesion and indirect structural and functional disconnection. Brain J. Neurol..

[b0245] Schaechter J.D., Fricker Z.P., Perdue K.L., Helmer K.G., Vangel M.G., Greve D.N., Makris N. (2009). Microstructural status of ipsilesional and contralesional corticospinal tract correlates with motor skill in chronic stroke patients. Hum. Brain Mapp..

[b0250] Shafto M.A., Tyler L.K., Dixon M., Taylor J.R., Rowe J.B., Cusack R., Calder A.J., Marslen-Wilson W.D., Duncan J., Dalgleish T., Henson R.N., Brayne C., Matthews F.E. (2014). The Cambridge Centre for Ageing and Neuroscience (Cam-CAN) study protocol: a cross-sectional, lifespan, multidisciplinary examination of healthy cognitive ageing. BMC Neurol..

[b0255] Siegel J.S., Ramsey L.E., Snyder A.Z., Metcalf N.V., Chacko R.V., Weinberger K., Baldassarre A., Hacker C.D., Shulman G.L., Corbetta M. (2016). Disruptions of network connectivity predict impairment in multiple behavioral domains after stroke. Proc. Natl. Acad. Sci..

[b0260] Souter N.E., Wang X., Thompson H., Krieger-Redwood K., Halai A.D., Lambon Ralph M.A., Thiebaut de Schotten M., Jefferies E. (2022). Mapping lesion, structural disconnection, and functional disconnection to symptoms in semantic aphasia. Brain Struct. Funct..

[b0265] Sperber C., Griffis J., Kasties V. (2022). Indirect structural disconnection-symptom mapping. Brain Struct. Funct..

[b0270] Stinear C.M., Barber P.A., Smale P.R., Coxon J.P., Fleming M.K., Byblow W.D. (2007). Functional potential in chronic stroke patients depends on corticospinal tract integrity. Brain J. Neurol..

[b0275] Tahmi M., Kane V.A., Pavol M.A., Naqvi I.A. (2022). Neuroimaging biomarkers of cognitive recovery after ischemic stroke. Front. Neurol..

[b0280] Taylor J.R., Williams N., Cusack R., Auer T., Shafto M.A., Dixon M., Tyler L.K., Cam-CAN H., R.n., (2017). The Cambridge Centre for Ageing and Neuroscience (Cam-CAN) data repository: Structural and functional MRI, MEG, and cognitive data from a cross-sectional adult lifespan sample. NeuroImage, Data Sharing Part II.

[b0285] Thiebaut de Schotten M., Foulon C., Nachev P. (2020). Brain disconnections link structural connectivity with function and behaviour. Nat. Commun..

[b0290] Umarova R.M., Beume L., Reisert M., Kaller C.P., Klöppel S., Mader I., Glauche V., Kiselev V.G., Catani M., Weiller C. (2017). Distinct white matter alterations following severe stroke: Longitudinal DTI study in neglect. Neurology.

[b0295] Varma S., Simon R. (2006). Bias in error estimation when using cross-validation for model selection. BMC Bioinformatics.

[b0300] Wawrzyniak M., Stockert A., Klingbeil J., Saur D. (2022). Voxelwise structural disconnection mapping: Methodological validation and recommendations. NeuroImage Clin..

[b0305] Weaver N.A., Kuijf H.J., Aben H.P., Abrigo J., Bae H.-J., Barbay M., Best J.G., Bordet R., Chappell F.M., Chen C.P.L.H., Dondaine T., van der Giessen R.S., Godefroy O., Gyanwali B., Hamilton O.K.L., Hilal S., Huenges Wajer I.M.C., Kang Y., Kappelle L.J., Kim B.J., Köhler S., de Kort P.L.M., Koudstaal P.J., Kuchcinski G., Lam B.Y.K., Lee B.-C., Lee K.-J., Lim J.-S., Lopes R., Makin S.D.J., Mendyk A.-M., Mok V.C.T., Oh M.S., van Oostenbrugge R.J., Roussel M., Shi L., Staals J., Valdés-Hernández D.C., M., Venketasubramanian, N., Verhey, F.R.J., Wardlaw, J.M., Werring, D.J., Xin, X., Yu, K.-H., van Zandvoort, M.J.E., Zhao, L., Biesbroek, J.M., Biessels, G.J., (2021). Strategic infarct locations for post-stroke cognitive impairment: a pooled analysis of individual patient data from 12 acute ischaemic stroke cohorts. Lancet Neurol..

[b0310] Werring D.J., Frazer D.W., Coward L.J., Losseff N.A., Watt H., Cipolotti L., Brown M.M., Jäger H.R. (2004). Cognitive dysfunction in patients with cerebral microbleeds on T2*-weighted gradient-echo MRI. Brain J. Neurol..

[b0315] Yeh F.-C., Panesar S., Barrios J., Fernandes D., Abhinav K., Meola A., Fernandez-Miranda J.C. (2019). Automatic Removal of False Connections in Diffusion MRI Tractography Using Topology-Informed Pruning (TIP). Neurother. J. Am. Soc. Exp. Neurother..

[b0320] Yeh F.-C., Tseng W.-Y.-I. (2011). NTU-90: a high angular resolution brain atlas constructed by q-space diffeomorphic reconstruction. NeuroImage.

[b0325] Zhao Y., Cox C.R., Lambon Ralph M.A., Halai A.D. (2022). Using in vivo functional and structural connectivity to predict chronic stroke aphasia deficits. Brain.

[b0330] Zhu L.L., Lindenberg R., Alexander M.P., Schlaug G. (2010). Lesion load of the corticospinal tract predicts motor impairment in chronic stroke. Stroke.

